# Blockchain and Artificial Intelligence Technology for Novel Coronavirus Disease 2019 Self-Testing

**DOI:** 10.3390/diagnostics10040198

**Published:** 2020-04-01

**Authors:** Tivani P. Mashamba-Thompson, Ellen Debra Crayton

**Affiliations:** 1 Department of Public Health, University of Limpopo, Polokwane, Limpopo Province 0727, South Africa; 2 Genesis Technology and Management Group, (GenesisTMG, LLC), Bethesda, MD 20817, USA; Ellen.Crayton@genesistmg.com

**Keywords:** self-testing, novel coronavirus disease-19, blockchain, artificial intelligence

## Abstract

The novel coronavirus disease 2019 (COVID-19) is rapidly spreading with a rising death toll and transmission rate reported in high income countries rather than in low income countries. The overburdened healthcare systems and poor disease surveillance systems in resource-limited settings may struggle to cope with this COVID-19 outbreak and this calls for a tailored strategic response for these settings. Here, we recommend a low cost blockchain and artificial intelligence-coupled self-testing and tracking systems for COVID-19 and other emerging infectious diseases. Prompt deployment and appropriate implementation of the proposed system have the potential to curb the transmissions of COVID-19 and the related mortalities, particularly in settings with poor access to laboratory infrastructure.

The novel coronavirus disease 2019 (COVID-19) has now reached sub-Saharan Africa (SSA) with cases reported in more than 40 SSA countries. SSA health systems are already battling with poor health outcomes and high mortality rates linked to the unique quadruple (HIV, Tuberculosis and non-communicable diseases) burden of disease [1]. In addition, SSA’s dense communities, informal settlements and rural and resource-limited settings are at particular risk and are most vulnerable to the COVID-19 outbreak. These populations are underserved in terms of health services and have the potential to become to new COVID-19 epicenters. The global COVID-19 statistics show surprisingly low transmission rates and fewer deaths in resource-limited countries, particularly countries in Sub-Saharan Africa (SSA). While SSA’s young population and warm climate may put SSA at an advantage for coping with the COVID-19 outbreak [2], there is growing concern about the impact of COVID-19 co-infections among the people living with other immune-system-weakening conditions such as HIV, TB and diabetes and the struggling health system in resource-limited settings such as SSA countries [3,4]. 

There is a growing concern about a failure to find and report cases, especially given weak health systems, inadequate surveillance, insufficient laboratory capacity and limited public health infrastructure in African countries [5]. Access to accurate diagnosis, monitoring and reporting of health outbreaks requires a well-resourced healthcare system [6]. Evidence shows that most resource-limited countries lack an effective, rapid surveillance system [7]. These settings also have a limited availability of health technologies for the electronic surveillance of infectious diseases to facilitate the prevention and containment of emerging infectious diseases such as COVID-19 [7]. Universal health coverage, access to high-quality and timely pathology and laboratory medicine (PALM) services is crucially needed to support health-care systems that are tasked with achieving Sustainable Developmental Goals [8]. This calls for the rapid development and deployment of health innovations for accurate diagnosis and electronic surveillance of COVID-19 in underserved populations. 

Recent evidence shows that prompt development and deployment of point-of-care (POC) diagnostics for screening in response to the COVID-19 outbreak can help to curb the spread of the disease and to alleviate the burden on the health system [9,10]. The impact of rapid testing on the COVID-19 death rate has been shown in Germany [11]. Emerging health innovations such as blockchain and artificial intelligence (AI) technology can be coupled with POC diagnostics to enable self-testing of patients in isolation as a result of exposure to COVID-19. Blockchain is a digital, public ledger that records online transactions. It involves the digital distribution of ledger and consensus algorithms and eliminates all the threats of intermediaries [12,13]. One of the commonly-known applications of blockchain is the crypto-currency Bitcoin [14], which has been successfully used as an alternative financial sector in emerging economies including countries in SSA [15]. Blockchain technology has shown adaptability in recent years leading to its incorporation in a wide range of applications including biomedical and healthcare systems [16,17,18]. The use of blockchain and AI in healthcare is evident in the following areas: management of electronic medical records; drugs and pharmaceutical supply chain management; biomedical research; education; remote patient monitoring; and health data analytics [17]. 

Mobile connected point-of-care diagnostics and self-testing has been successfully implemented in resource-limited settings [19,20,21]. However, there is limited evidence on the use of blockchain and AI technology for disease diagnosis. Bearing in mind the era of COVID-19 and the evidence on the overburdened healthcare systems and poor disease surveillance systems in resource-limited settings, and taking advantage of the available mobile Health (mHealth) systems, we recommend, a rapid development and deployment of low cost blockchain and AI-coupled mHealth connected self-testing and tracking systems as one of the strategic response strategies for COVID-19 and other immerging infectious diseases (Figure 1).

The initial step for this system is through a mobile phone or tablet application (app) which could be adapted from existing self-testing apps [22,23]. The app will request a user’s personal identifier before opening pre-testing instructions. Following testing, the user will upload results into the app. The blockchain and AI system will enable the transfer of the test result to alert the outbreak surveillance authorities of all tests performed as well as the number of positive and negative test results. This will help ensure that all positive cases are referred to a quarantine site for treatment and monitoring. The in-built geographic information system (GIS) in mobile devices will enable the tracking of the people who tested positive. This system will also be connected to the local and international databases to ensure appropriate surveillance and control of the outbreak.

The AI component of this technology will enable potent power in data collection (patient information, geographic location of the patient and test results), security, analysis, and curation of disparate and clinical data sets from federated blockchain platforms to derive triangulated data at very high degrees of confidence and speed. With this well-architected integrative technology platform, we assure secure and immutable data sets that enable the collection of high-quality data and can draw deep insights. Local development of these diagnostics can help overcome the supply chain challenges [24] and the cost which can limit accessibility of POC diagnostics in resource-limited settings. This technology can be adapted for use in community-based case finding of other infectious diseases such as HIV, TB and Malaria, which may be exacerbated by the current COVID-19 outbreak. Relevant stakeholders’ involvement will be crucial to ensure the efficient development and sustainable implementation of the proposed technology, particularly in underserved populations.

## Figures and Tables

**Figure 1:**
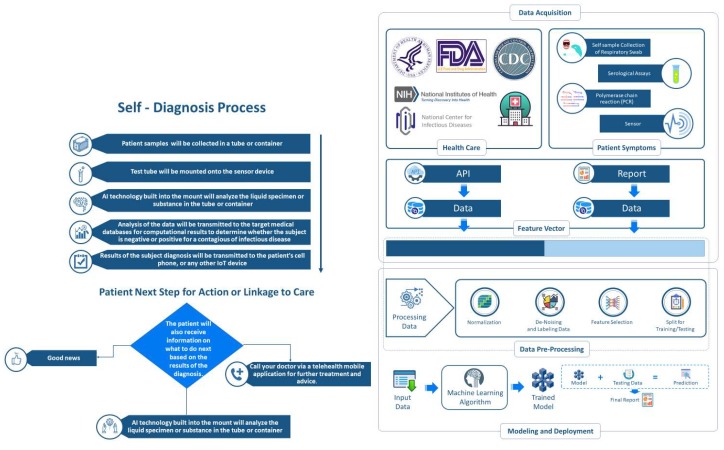
Proposed community-based blockchain and artificial intelligence-coupled mobile-linked self-testing and tracking system for emerging infectious diseases.
